# Redefining organizational digitality: a relational-ontological approach inspired by new materialism

**DOI:** 10.3389/fsoc.2024.1426930

**Published:** 2024-10-25

**Authors:** Linda Maack

**Affiliations:** Arbeitsbereich Organisationspädagogik, Fachbereich Erziehungswissenschaften und Psychologie, Freie Universität Berlin, Berlin, Germany

**Keywords:** organizational digitality, relational onotology, intra-action, apparatus, new materialism, organizational orders

## Abstract

Organizations, as central actors in societal structure, undergo significant transformations due to the impact of digitalization, often resulting in disruptive changes. Consequently, organizations increasingly view digitalization as an ongoing process of negotiation, which has led to the emergence of new operational modes and organizational norms. In this context, the interaction between organizations and digital technologies is characterized by recursive dynamics, which blur conventional boundaries. This presents a challenge in defining the distinct domains of the digital and the organizational within the framework of recursivity. This article draws upon new materialism and agential realism to propose an ontological-relational approach to understanding organizational digitality. This approach suggests a reconceptualization of organizational digitality as a mechanism that generates relational entities, thereby reshaping their inherent meanings. By transcending traditional boundaries between organizations and digital, this perspective provides a nuanced understanding of digital phenomena within organizational contexts.

## 1 Introduction

The process of digitalization is not merely the transfer of analog to digital processes or the introduction of digital tools. Rather, it is a constitutive and altering process at the societal, institutional-organizational, and interactive-individual levels, causing responsibilities to shift and be (re)arranged. Organization understood as the process of organizing as well as the resulting entity (King et al., 2010) are strongly affected by digital transformation processes. By digitizing various processes, including organizational ones, digital media and technology are not only used to outsource various actions to digital tools or carry them out, but these new processes also lead to new organizational configurations and structures (Allert et al., 2017). Digital transformation processes are becoming integrated into organizations, sometimes leading to disruptive changes (Wendt, 2021; Ahrens, 2022). Consequently, organizations increasingly view digitalization processes as recursive negotiation processes that lead to the development of new modes of action and organizational practices, rather than as unidirectional (Truschkat et al., in preparation). From an organizational perspective, digitalization presents an opportunity for structure-forming and culture-creating processes, that can also produce new organizational practices (Kuusisto, 2017; Bernhard-Skala, 2021; Keller et al., 2021). Accordingly, initial approaches in this direction acknowledge that organizations are not merely passive recipients of digital technology, but rather active creators of digital phenomena (Büchner, 2018; Kirchner, 2019). In accordance with this, the organization is here defined as an active player that is able to interact with the digital and steer and shape corresponding transformation processes. This recursive interrelationship between organization and the digital, understood to be between two delimitable entities, is posited as the basis of this relationship. The concept of organization and digitality is therefore defined as two actors that produce digitality in a joint interaction, while maintaining their entity status throughout the process. However from this point, attempting to conceptualize the digital and the organizational as discrete entities presents a significant challenge in itself, when recursivity fundamentally undermines this demarcation. It can thus be questioned as to whether and to what extent boundaries between the digital and the organizational are reinforced or dissolved within the context of recursivity.

The theoretical currents of new materialism intend to advance the perspective of capturing this specific point of view. New materialism posits that greater attention should be paid to the materiality and objects when considering society and its transformation. At the core of this examination lies the premise of a co-constitutive generation of reality, wherein matter is not conceived as passive, but rather as an agent engaged in the production of knowledge and reality (Coole and Frost, 2010). In this context, materiality can be employed as a novel factor in the transformation and learning processes within organizations. Barad (2003, 2007) theory of agential realism, which aligns with the tenets of new materialism, is predicated on a relational ontology and challenges anthropocentric worldviews and dualistic systems of thought. The focus here is on the interplay between meaningful-symbolic and material orders, wherein entities are not only understood beyond their object status, but also conceptualized as produced through intra-action (Tuin and Dolphijn, 2012; Hoffarth et al., 2023). From an analytical perspective, it is therefore about the dissolution of boundaries between the human and the non-human and their co-constitutive potential.

The article posits that previous conceptualizations of the interrelationship between organizations and digitalization in Germany are inadequate for analytically identifying the extent of the dissolution of boundaries in digitality. This is particularly evident in the absence of integration of the international discourse on sociomateriality (Section 2). Therefore, with the help of new materialism and in particular Barad (2003, 2007) agential realism, the interrelationship between the organization and the digital can be understood as relational-ontological (Section 3). This enables the formulation of an alternative proposal for the conceptualization of organizational digitality. In this model, organizational digitality is conceived as an apparatus, according to Barad, which generates particular relata, here organizational and digital actors, in terms of their initial meanings (Section 4). The article concludes by suggesting the potential of an ontological-relational perspective on digitality in the organization within German organizational research (Section 5).

## 2 Rethinking the interplay of organization and digitality

In recent years, there has been a stronger focus on the interaction and relationality between organizational and digital processes, even though digitalization was not previously considered in the context of organizations or as organization-neutral (Büchner, 2018; Wendt, 2020; 2021; Kette and Tacke, 2022). Digitalization processes of, within and between organizations are increasingly no longer understood solely as a unidirectional process (Büchner, 2018) in which digitalization is simply introduced into the organization or analog processes are merely translated into digital. Instead, they are seen as a recursive negotiation process (Truschkat et al., in preparation). The concept of digitalization, which primarily concerns the implementation of digital technologies or the conversion of analog to digital media, cannot be reduced to technocentric inquiries about accessibility, usability, and restructuring alone (Mayrberger, 2020). Accordingly, there is a growing understanding that views digitalization as a bidirectional process that involves both the introduction of technologies and the organizational framework. This reciprocal construction process means that not only does digitalization transform the organization, but the organization itself carries out digitalization (Büchner, 2018; Büchner and Dosdall, 2022). The term “digitality,” as defined by Stalder (2016), also plays a pivotal role in this increased focus on viewing digitalization processes as interactive within organizations. In contrast to the technocentric concept of digitalization, Stalder (2016) posits the concept of digitality, which he defines as a structure of cultural practice patterns that are produced both in routinized actions with digital media and have an action-oriented effect on (organizational) actors. In the context of digitality, technologies become the primary reference point for human action, while digital practices also serve as a fundamental frame of reference within the contextualization, execution, and evaluation of actions (Allert et al., 2017). Although the provision of a digital infrastructure by the organization is the basis for what can be considered digitality, following this understanding, it is primarily produced in a socio-technical process of interaction between various digital and non-digital entities (Maack and Vollmar, 2023).

The findings of previous research and theoretical works on the relationship between organizational and digital structures in Germany follow this common assumption and consideration: the recursive interrelationship between these two entities. These studies posit that digitality is the result of a two-way interaction between the organization and digital artifacts, where both entities are active agents in this process and maintain their distinct entity statuses despite this interaction. For example, Dörner and Rundel (2021) posit that organizations are active players in the digital transformation and illustrate this using conceptual patterns of how organizations deal with digitalization. In this view, organizations are seen as active players in the digital transformation. The organization is attested to have the capacity to act, which becomes particularly evident in the argumentation about the various approaches to dealing with active change or active refusal in and with digitalization. Although a recursive negotiation process is described here, the emphasis on the organization’s active engagement with digital artifacts means that it is understood as a confronting entity. Consequently, the negotiation is conceived as relational, yet also binary. This line of inquiry can be extended to studies of algorithm-based decision-making processes in organizations. Kette (2021) notes that the algorithmizing of decision-making processes in organizations does not necessarily result in a loss of importance for organizations, but rather that organizations gain decision-making autonomy. Similarly, Wendt and Manhart (2020) emphasize that the implementation of organizational decisions based on algorithmic technologies makes personal decisions, which are organized by the organization as an actor, even more important. Even if relational negotiation of decisions is again referenced in this context, the primary focus is on the organization as a decision-making and acting entity, with algorithms. While the significance of algorithms is constituted in their interaction with the organization and is therefore essentially entity-less prior to that interaction, they are nevertheless designed as a counterpart and independent factor in organization decision-making processes.

As it shows these exemplary studies have placed particular emphasis on the recursive negotiation between digital technologies or artifacts and the organization as the constitution of new digital practices. In this relational process, however, the organization has been placed at the center of the analysis, as it is viewed not only as a purely functional regulatory framework, but also as a structuring element of situated action. Consequently, the organization is regarded as a key agent in the digitalization process, operating within the context of this interrelationship. Although the recursive nature of the negotiation process is acknowledged here, the organization and digital technologies are regarded as two distinct entities that interact in a relational process. However, the concept of digitality can be viewed not as an entity in and of itself, but rather a phenomenon created through interaction. This suggests that the term “organizational digitality” may be more accurate (Vollmar and Maack, in preparation). Such a perspective naturally prompts the question of a potential theoretical framework that not only challenges this dichotomous delimitation and selectivity, but also eliminates it.

The sociomateriality paradigm (Leonardi, 2012; Carlile et al., 2013), which is discussed and used in the international context of organization theory, examines the inseparable interweaving of social and material aspects in organizations, digital technologies, and everyday practices. The term emphasizes that social and material elements do not exist separately but are intertwined in a complex network of relationships. Theories within the sociomaterial category are derived from various theoretical foundations and encompass a range of approaches and perspectives (Faulkner and Runde, 2012). These theories posit that the construction and alteration of daily practices occurs through the ongoing interaction between human activities and non-human factors. They further assert that sociomaterial elements create a diverse, integrated whole comprising technical, natural, and cognitive components. Additionally, they posit that both humans and non-humans are the products of networks of connections and activities, which are all based on a complex web of relationships (Moura and Bispo, 2019). Fenwick et al. (2011) identify four key theories that fall under the sociomaterial category: actor-network theory (ANT), cultural-historical activity theory, complexity theory, and spatial theory. In addition to these, Moura and Bispo (2019) propose that four additional approaches can also be regarded as sociomaterial, including organizational aesthetics, science and technology studies (STS), ANT i-History, and new materialities (*ibid.*). Despite their prevalence in international discourse on organizations and technology (Orlikowski, 2007; Leonardi, 2012, 2013; Orlikowski and Scott, 2015), as well as the increasing relevance on digitality (Cecez-Kecmanovic et al., 2014; Bader and Kaiser, 2017), these theories have been relatively underrepresented in the German discourse to date. The only exceptions are on the one hand Bader (2020) work on “Human-technology work.” Her work examines the intricate interrelationship between human actors and technical systems in the context of work processes and organizations. It underscores the interdependence of humans and technology, with both continuously adapting to changing conditions. On the other hand, studies employing a network theory approach, such as those using the Actor-Network Theory (ANT) methodology or the concept of assemblage. The ANT methodology is particularly interested in the involvement of non-human actors, called “actants,” in the formation of networks and, consequently, of particular interest for digital artifacts (Pätzold, 2017; Pätzold and Bestvater, 2019). ANT emphasizes the significance of things and provides a theoretical sharpening of action on those actants for and on organizational processes and developments (Pätzold, 2017). Similarly, organizations are discussed as intricate elements of algorithmic assemblage in Büchner et al. (2024). In this context, the concept of an assemblage, understood as a socio-material network, is interpreted in terms of power theory. This implies that algorithmic regimes are to be understood as socio-technical assemblages of knowledge production and dissemination. Therefore, from an organizational perspective, there is a need to examine organizations as specific and complex elements of these algorithmic assemblages.

In a reiteration of the previous point, the capacity of the organization to act independently is once again highlighted in these studies, this time within the context of network theory. However, the organization itself is conceived as an entity that functions as part of a network, engaging in a co-constitutive creative process. Therefore, this networking of actors (or actants) represents a relational, but not an ontological understanding of digitality. Nevertheless, in a sociomaterial understanding, the concept of digitality within organizational contexts is not considered an independent entity. Rather, it can be defined as a phenomenon that arises from the interactions and relationships within the organization. From this perspective, attempting to understand digital artifacts or technologies and the organization as two distinct entities is a challenging, if not an impossible task. This raises the question of how we may distinguish and separate the digital and the organizational, given that recursivity actually removes this boundary. As an example, the act of composing an email or utilizing a digital application can be understood to represent both an organizational and a digital process. Alternatively, the integration of collaboration tools (e.g., Slack, Microsoft Teams, or Trello) facilitates the convergence of social and material aspects by transforming the manner in which employees communicate and collaborate. However these tools not only facilitate novel forms of social communication, but also engender the emergence of novel organizational workflows and processes situated at the nexus between the organizational and the digital, that did not exist before. An additional example is the use of chatbots in customer service departments, which integrates AI technology with human interaction. Chatbots process routine inquiries and forward more complex requests to human employees, thereby altering the dynamics of customer interactions and work processes. The interplay between customers and employees with AI technologies gives rise to a novel form of digital communication that straddles the divide between analog and digital. The examples presented illustrate the concept of sociomaterial interdependence, which can be defined as the entanglement of different materialities that continuously co-construct reality while at the same time co-constituting each other. Therefore it remains unclear where the boundaries are set and “who” produces “whom” here. Rather, a new phenomenon emerges that dissolves precisely these boundaries.

In light of the preceding assumptions, the necessity of a theoretical framework emerges ones more, as social materiality may be more effectively understood as a paradigm than a theory. Despite the fact that it challenges the dichotomous delimitation and selectivity that has been previously identified, and eliminates it. In this context, a neo-materialist perspective, which is one of the theoretical frameworks of a sociomaterial understanding of digitality, can provide an extension or focus on precisely this co-constitutive moment by analyzing the dissolution of boundaries and dichotomies between objects, things, and materiality, as well as its co-constitutive productions. This theoretical framework has not only been and continues to be vigorously debated across various disciplines (see, for instance, Bettinger, 2020 on media education and Hoffarth et al., 2023 on educational science, and Wanka and Gallistl, 2018 on Socio-Gerontechnology), but has also been used in the international context of organizational studies by Orlikowski (2007), Orlikowski and Scott (2008, 2015) or de Vaujany et al. (2024) among others. Nevertheless there has been little engagement with it in the German context of organizational studies.

## 3 Relational ontology of digitality

New materialism represents a perspective within the social sciences that advocates for a greater focus on matter and materiality (Barad, 2003, 2007; Bennett, 2010). At the core of this approach is the interplay between the sensual-symbolic and material orders, wherein matter is understood to exist beyond its purely objective status. Rather, matter is ascribed a form of agency and a correspondingly strong effect on human actors within the joint interaction (Hoppe and Lemke, 2021). Despite the diversity in the studies, there is a commonality in the assumption of a co-constitutive emergence in which matter is not understood as passive but as a “partner” or “active constructor” involved in the production of knowledge and reality (Wiertz, 2021). Following these considerations, the question of materiality of the digital (Wunder, 2020; Leineweber et al., 2023a) and the relationship between materiality and digitality (Bettinger, 2020; Unterberg, 2023; Leineweber et al., 2023b) is increasingly being negotiated as a significant issue in this field. Nevertheless, contemporary discussions have concentrated on the fluidity and softening of the dichotomy between analog and digital (Bettinger, 2020; Wunder, 2020), as well as the associated consideration of materiality as a dynamic and action-oriented factor in the production of reality. With regard to the processes of digital transformation, the question of the significance and participation of digital technology within the production of knowledge and reality appears to be particularly relevant, given the strong organizational changes and reorganizations that technology, media, and digitality in particular bring about. This idea was particularly advanced by Orlikowski (2007) and Orlikowski and Scott (2008) in the context of organizations, through a debate about the constitutive entanglement between the social and technical elements in organizations. The concept of constitutive entanglement does not seek to privilege any one element—human, technological, or otherwise—nor does it attempt to explain the relationship between the two. Instead, it considers them to be inextricably linked (Orlikowski and Scott, 2008). From this perspective, agency can be attributed to the digital, and it should firstly be understood in this context as a phenomenon that arises through the recursive interplay or co-constitution of various digital and analog actors, and secondly, materializes in the process of drawing boundaries between the analog and the digital.

These considerations will be developed further below. Particular attention is paid here to Barad (2003, 2007) agential realism and their concept of apparatus and intra-action, which focuses on this intra-active creative process of digitality. Karen Barad’s theory of agential realism is considered one of the most influential schools of thought within new materialism and has been the subject of considerable interest in the international debate on organization and materiality (Orlikowski and Scott, 2015; Moura and Bispo, 2019). However, in order to be able to focus on the productivity of drawing boundaries between analog and digital in the joint intra-action following Karen Barad, it is necessary to briefly outline the cornerstones of their developed theory of agential realism (see in detail Coole and Frost, 2010; Tuin and Dolphijn, 2012). Barad (2012) bases their theory on the criticism that “language […] has been granted too much power” (*ibid.*: 7) and advocates for a shift towards materiality. However, Barad is not only concerned with a re-centring of materiality. What is equally important is that, despite their emphasis on materialities, they also present a relational ontology that rejects the priority of the relata over the relation. For Barad, the existence of objects (relata) can only be produced by relations, which is called phenomena: “That is, phenomena are ontologically primitive relations - relations without preexisting relata” (Barad, 2007, p. 139). In their work, Barad draws on the theories and concepts of Michel Foucault, Judith Butler, and quantum physicist Niels Bohr. However, reinterprets them through a posthumanist lens, as demonstrated in their concept of the phenomenon.

The concept of the apparatus (with recourse to Niels Bohr and Donna Harraway) is particularly prominent, although it is not limited to physical experiments in the laboratory. According to Barad, apparatuses are material-discursive references or, more generally, practices that draw boundaries and generate meanings and properties by measuring or observing (Tuin and Dolphijn, 2012). Therefore, apparatuses in Barad’s work can be seen as boundary-drawing practices, which can include social, non-scientific, and everyday practices (Coole and Frost, 2010). Apparatuses function as border-drawing practices, creating a separation between subject and object, as well as other dualisms that exist within our surroundings. Barad uses the term “agential cut” or, in their earlier work, “constructed cut” to describe the constructed nature of boundaries, particularly between subject and object. Nyckel (2022) suggests that “constructed cut” better captures the essence of this concept. In addition to the constructed nature of boundaries, the agential cut also constructs and produces corresponding meanings and properties (within the respective phenomenon) (*cf. ibid.*). For this concept of contructedness, production, or emergence of relations through the common relation, Barad introduces a further term: intra-action. This distinguishes it from the concept of interaction, which presupposes the existence of independent and self-contained entities, whereas intra-action emphasizes the mutual production. “The relata in the world thus do not interact in agential realism by meeting in the world, they intra-act in the sense that they are always constituted in intra-actions” (Nyckel, 2022, p. 190). This emphasis on questioning and dissolving boundaries is a key aspect of Barad’s theory, as demonstrated by the concept of intra-action, which challenges fixed categories and boundaries (Hoppe and Lemke, 2015). The concept of dissolving known boundaries leads to their new understanding of materiality as inherently dynamic and involved in holistic production processes. “Matter’s dynamism is generative not merely in the sense of bringing new things into the world but in the sense of bringing forth new worlds, of engaging in an ongoing reconfiguring of the world” (Barad, 2007, p. 170). Matter has its own dynamic and is actively involved in the constitution of the world. Barad refers to this independent dynamic as agency, which is related to the German concept of agency (dt. Handlungsfähigkeit). For them, agency is a dynamic concept that is defined in terms of the theory of action or solely in terms of the possibility of action and not as a possession or property.

“Crucially, agency is a matter of intra-acting; it is an enactment, not something that someone or something has. It cannot be designated as an attribute of subjects or objects (as they do not preexist as such). It is not an attribute whatsoever. Agency is “doing” or “being” in its intra-activity” (Barad, 2003, p. 827).

Agency is linked to intra-action and can be understood as “doing” in reference to Butler’s concept of performativity in a post-humanist context, or as a “performative reciprocal relationship.” This perspective is based on an understanding of agency that does not conceive of it as a human ability, but rather as material-discursive (re-)configurations of the world in which non-humans are also involved. The resulting phenomena constitute reality in the first place (Eickelmann, 2022). In regard of (digital) technology performativity, Orlikowski and Scott (2008) posits that it does not exist in a prior state; rather, it is produced through social practices. Such an understanding requires a grasp of how technological components affect the generation of meaning and human conduct, as well as how they are themselves influenced (Orlikowski, 2007). Therefore, Barad’s theoretical framework and terminology, particularly the concept of apparatus, can be utilized to analyze the phenomenon of organizational digitality. This analytical lens enables a nuanced understanding of the co-constitutive production process within organizational digitality.

## 4 Organizational digitality as apparatus

As has already been made clear above, the assumption of a recursive negotiation process between organizations and digitalization is clearly evident in the first empirical studies and concepts. In these studies, it is evident that organizations are not merely passive recipients of the digital; they are also active agents of change (Büchner, 2018). The majority of studies in Germany place the organization at the center of their analysis and demonstrate how digitalization occurs as a negotiation process between the organization and digital artifacts, as exemplified by algorithm-based decision-making in organizations (Wendt and Manhart, 2020; Egbert et al., 2022). Consequently, the organization and the digital are conceived as two opposing entities that engage in a recursive negotiation process in the sense of an interaction, but which exist in advance of this interaction. At this juncture, an ontological relational perspective can be adopted with the assistance of Barad’s agential realism, which builds upon the prevailing recursive understanding of digitality but extends it to encompass the ontological aspect. If the digital and the organizational merge relationally in the process of digitality, then the supposed boundaries between them can be questioned and the processes of boundary-drawing can be taken into consideration. A relational-ontological perspective on organizational and digital actors suggests that both entities (relata) emerge only in the joint intra-action (relation) and simultaneously produce the phenomenon of digitality. In this context, organizational digitality can be understood, with recourse to Barad, as a (relational) phenomenon and at the same time an apparatus, which only emerges through the intra-action and boundary setting of organizational and digital actors in the sense of an intra-activity of becoming. The concept of organizational digitality as apparatuses implies that they facilitate agential or constructed cuts and are thus a fundamental element of the resulting phenomenon. It is important to recall that Barad’s apparatuses are boundary-drawing practices in general, which generate meanings, boundaries, and properties of and in phenomena through measurements and observations. In light of this understanding, organizational digitality can be understood as an apparatus which performs certain constitutive cuts. These cuts serve to define and give meaning to the phenomenon itself, as well as to specific elements within the organization. Furthermore, they delineate boundaries and establish order within this organizational context. This perspective is also possible because, in general, orders in organizations refer to the regular, emphasizing the underlying structure that gives regularity its stability. This structure is addressed in a special way, as it is the source of the regularity’s permanence or materialization (Truschkat et al., 2017). In this manner, orders are established as a consequence of negotiations regarding the objects in question, or alternatively, the objects themselves become the subject of negotiation. Consequently, orders are manifested in organizational practice (*ibid.*). Following this understanding, organizational digitality, as defined by Barad as boundary-drawing practices, brings forth certain relations in their meaning in the first place. In this case, the relations in question are those between digital and organizational actors. Consequently, the actor is constituted in its specific meaning as an organizational actor through organizational digitality, and the digital medium is produced in its capacity as a digital actor through organizational digitality. Concurrently, it is within the context of the intra-action between digital and organizational actors that organizational digitality assumes its significance. Consequently, organizational digitality functions as an apparatus that continuously constitutes both the organizational and digital actors, as well as the phenomenon of organizational digitality itself.

For example, an organization implements a new project management software. The existence, significance and boundary between the different entities (including the digital software and the employees) is only established through their joint intra-action. The software delineates tasks, deadlines, and responsibilities, while employees adapt their work habits to the software’s functionalities. At the same time this software acts as an apparatus facilitating agentic cuts. These divisions serve to establish and give meaning to various aspects of the project management process, such as defining what constitutes a completed task or a project milestone. By organizing tasks and deadlines, the software creates boundaries and generates meanings for those boundaries, such as the distinction between tasks assigned to different departments. This boundary-drawing is a dynamic process where the software continuously influences how employees perceive and intra-act with their work. Over time, these practices become regular and stable, forming the underlying structure of project management in the organization. Through these intra-action and boundary-drawing practices, the software and its use within the organization define the roles and identities of both digital and organizational actors. The roles and workflows of employees are shaped by their intra-actions with the software, while the software’s role as a project management tool is defined by its use within the organizational context. It can be argued that the software, in its continual process of shaping and being shaped by its intra-action with employees, constitutes the phenomenon of organizational digitality. It facilitates the maintenance of order and structure within the organization through its boundary-setting practices. In conclusion, organizational digitality, in the context of Karen Barad’s framework, is a dynamic and relational apparatus and phenomenon that emerges from the continuous intra-action between digital tools and organizational actors.

This illustrative example serves to demonstrate that this apparatus is a complex entity, comprising a multitude of interrelated intra-actions and borderline practices. However it becomes evident that a clear distinction between phenomena and apparatus is not always possible, since the concept of organizational digitality as an apparatus ultimately results in the production of entities and phenomena. However, this is also where the benefit of this perspective lies, since apparatuses as well as phenomena can be understood fluidly and without external boundaries, and are always intertwined with other apparatuses. In accordance with Barad’s theory, borders and barriers do not exist in a state of isolation; they are neither inherently benign nor impartial, but have been constructed and reinforced in dependence on other apparatus and practices (Coole and Frost, 2010). Applied to the domain of organizational digitality, this implies that border-crossing practices cannot be viewed in isolation; rather, they are contingent upon the existence of other (organizational) practices and orders. They are thus the fundamental way in which the organization realizes itself.

## 5 Conclusion

The objective of this paper was to capture the recursiveness of the relationship between organization and digitality within a neo-materialist approach, taking into account Barad’s concept of agential realism. It became evident that the relationship between organization and digitality can be primarily conceptualized as a recursive negotiation process. The majority of empirical studies and theoretical concepts, that are discussed in the German discourse, highlight the active role of organizations in this process, demonstrating that they are not merely passive recipients but also agents of digitalization. Although this emphasizes the relationality of digitality in the organization, it refers to two pre-existing entities. This distinction, which is effectively negated by the relationality, should therefore be reconsidered with regard to the article. In order to incorporate this perspective, the recursive nature of organization and digitality was considered from the perspective of Karen Barad’s relational-ontological theory, which is situated within the international discourse of sociomateriality. This illustrates how the growing incorporation of digital technologies into organizational procedures creates new forms of materiality that transcend traditional material and immaterial boundaries. It became evident that digital technologies cannot be regarded as mere tools, but rather as active agents that shape and influence organizational realities. In accordance with the theoretical framework proposed by Karen Barad, both organizations and digital technologies emerge through their joint intra-action. Digitality is therefore understood as a relational phenomenon that emerges through the boundary setting and intra-action of organizational and digital actors. This was exemplified by examining organizational digitality as a powerful apparatus. This is because organizational digitality makes constitutive cuts that produce both specific actors in their significance and ultimately digital phenomena in the organization. Consequently, the phenomenon of organizational digitality emerges only as a result of the intra-action between digital and organizational actors. The significance of this perspective lies in its negation of the existence of relata prior to the relation. The article thus presents an opportunity to view organizational digitality as an apparatus, rather than merely a tool, and as an active and constitutive component of organizational reality. A reevaluation of organizational structures, processes, and decision-making mechanisms is required to consider the possibilities and opportunities inherent in this perspective. A relational-ontological perspective on organizational digitality presents novel challenges, yet also offers potential perspectives to the German discourse on digital organizations that transcend conventional notions of materiality and organization. In addition to the significance of digitalization issues, the dissolution of boundaries and restructuring that is associated with it can also be examined more closely from this perspective.

## Data Availability

The original contributions presented in the study are included in the article/supplementary material, further inquiries can be directed to the corresponding author.
